# Identifying biomarkers related to motor function in chronic stroke: A fNIRS and TMS study

**DOI:** 10.1111/cns.14889

**Published:** 2024-07-28

**Authors:** Guiyuan Cai, Jiayue Xu, Cailing Zhang, Junbo Jiang, Gengbin Chen, Jialin Chen, Quan Liu, Guangqing Xu, Yue Lan

**Affiliations:** ^1^ Department of Rehabilitation Medicine School of Medicine, The Second Affiliated Hospital, South China University of Technology Guangzhou China; ^2^ Guangzhou First People's Hospital, Guangzhou Medical University Guangzhou China; ^3^ Postgraduate Research Institute, Guangzhou Sport University Guangzhou China; ^4^ Department of Rehabilitation Medicine Guangdong Provincial People's Hospital (Guangdong Academy of Medical Sciences), Southern Medical University Guangzhou China; ^5^ Guangzhou Key Laboratory of Aging Frailty and Neurorehabilitation Guangzhou China

**Keywords:** biomarker, functional near‐infrared spectroscopy, machine learning, motor impairment, stroke

## Abstract

**Background:**

Upper limb motor impairment commonly occurs after stroke, impairing quality of life. Brain network reorganization likely differs between subgroups with differing impairment severity. This study explored differences in functional connectivity (FC) and corticospinal tract (CST) integrity between patients with mild/moderate versus severe hemiplegia poststroke to clarify the neural correlates underlying motor deficits.

**Method:**

Sixty chronic stroke patients with upper limb motor impairment were categorized into mild/moderate and severe groups based on Fugl‐Meyer scores. Resting‐state FC was assessed using functional near‐infrared spectroscopy (fNIRS) to compare connectivity patterns between groups across motor regions. CST integrity was evaluated by inducing motor evoked potentials (MEP) via transcranial magnetic stimulation.

**Results:**

Compared to the mild/moderate group, the severe group exhibited heightened premotor cortex–primary motor cortex (PMC–M1) connectivity (*t* = 4.56, *p* < 0.01). Absence of MEP was also more frequent in the severe group (*χ* ^2^ = 12.31, *p* = 0.01). Bayesian models effectively distinguished subgroups and identified the PMC–M1 connection as highly contributory (accuracy = 91.30%, area under the receiver operating characteristic curve [AUC] = 0.86).

**Conclusion:**

Distinct patterns of connectivity and corticospinal integrity exist between stroke subgroups with differing impairments. Strengthened connectivity potentially indicates recruitment of additional motor resources to compensate for damage. These findings elucidate the neural correlates underlying motor deficits poststroke and could guide personalized, network‐based therapies targeting predictive biomarkers to improve rehabilitation outcomes.

## Graphical abstract

Content of the study. First, functional near‐infrared spectroscopy and transcranial magnetic stimulation assessments were conducted, followed by feature extraction. Subsequently, independent *t*‐tests and chi‐squared tests were applied to reduce the feature dimensionality, and machine learning models were developed with the retained features. Finally, the performance of the models was evaluated by the area under the receiver operating characteristic curve and accuracy. Bayesian models effectively distinguished subgroups and identified the premotor cortex–primary motor cortex connection as highly contributory.

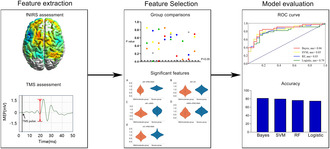

## 1 INTRODUCTION

Upper limb motor impairment is a common outcome of stroke, and it greatly inhibits the ability of patients to perform activities of daily living, impairing their quality of life.^1^ Recovery of poststroke upper limb motor impairment largely depends on brain plasticity, which can be defined as structural and functional adaptations of the neuronal elements and networks.^2^ Understanding the relationship between the characteristics of brain networks and poststroke motor function is important for developing optimal therapeutic strategies aimed at rehabilitation. Nevertheless, a comprehensive understanding of the neural mechanisms associated with motor impairment remains elusive.

The integrity of the corticospinal tract (CST) is considered a crucial determinant influencing motor function poststroke. A key method to assess functional integrity of CST involves using TMS to stimulate the primary motor cortex (M1) on the affected side and observing for the induction of motor evoked potentials (MEP). Studies have highlighted MEP status (MEP+ vs. MEP−) as a promising indicator of motor outcomes.^3^ Additionally, researchers have developed a predictive recovery potential (PREP) algorithm, integrating motor scores, MEP status, and MRI measurements, exhibiting strong predictive abilities for stroke patients' motor outcomes.^4^ However, in a recent study, researcher found that absence of MEP does not predict poor recovery in patients with severe stroke,^5^ indicating that relying solely on MEP status may not comprehensively capture the multifaceted nature of poststroke motor function recovery. This prompts the exploration of complementary indicators to provide a more holistic understanding.

In addition to CST integrity, functional neuroimaging has been utilized to ascertain the characteristics of cortical networks associated with motor function, revealing numerous proposed biomarkers, including interhemispheric functional connectivity (FC) of the M1.^6^, ^7^ However, the results of different studies have been inconsistent. While some studies found enhanced interhemispheric M1 connectivity,^6^ others reported no significant difference^8^ or lowered connectivity in stroke patients with deficits.^9^ These inconsistent results may be attributable to the influence of different severity of motor impairment. Indeed, poststroke reorganization of brain networks is heterogeneous, resulting in different degrees of upper limb motor recovery. In Liu et al.'s study, patients with partial hemiplegia exhibited a positive correlation between motor function and FC in sensorimotor circuits, while complete hemiplegia displayed contrasting correlations.^10^ Besides, study has shown that patients with mild hemiplegia predominantly engage the motor area on the affected side during movement, whereas those with severe hemiplegia involve a broader range of areas, including the unaffected hemisphere.^11^ Unraveling the reorganization patterns in patients with diverse severity of hemiplegia is crucial for devising targeted treatment strategies specific to each subgroup. However, the current investigation of brain function distinctions among these hemiplegic subgroups has been constrained to a limited number of studies, emphasizing the ongoing necessity for expanded research in this field.

So far, most previous studies have focused on activities of the essential cortical areas of the motor network, including M1, supplementary motor area (SMA), and premotor cortex (PMC).^12^, ^13^, ^14^ Interestingly, there are growing evidences to suggest that stroke is a disorder of brain networks involving regions beyond the motor network. In a recent study, interfering with the neural activity of the parietal cortex using transcranial magnetic stimulation (TMS) was found to modulate the motor performance of stroke patients, suggesting a contribution of the parietal cortex to the motor network.^15^ Moreover, Bönstrup et al.^16^ found a stronger coupling in the alpha bands between the parietal cortex and motor cortex in stroke patients compared to controls and the upregulation of parietofrontal network was positively related to motor function. Nevertheless, currently few studies have explored the relationship of parietal cortex and poststroke motor network. Therefore, more research is required to confirm their association.

Therefore, we aimed to investigate the mechanisms underlying poststroke brain reorganization by integrating TMS assessments and functional near‐infrared spectroscopy (fNIRS). In this current study, we stratified patients into mild/moderate and severe groups based on the Fugl‐Meyer assessment (FMA). Subsequently, we employed fNIRS to measure their brain network characteristics and assessed the functional integrity of the CST using TMS. Our investigation had two primary objectives. First, we aimed to compare resting‐state FC and CST integrity between the two stroke subgroups using fNIRS and TMS assessments, respectively. Second, we endeavored to develop machine learning models with the aim of identifying biomarkers associated with motor function.

## 2 MATERIALS AND METHODS

### 2.1 Participants

Participants were recruited using the following inclusion criteria: (1) first‐ever stroke confirmed by neuroimaging (CT or MRI); (2) age > 18 years; (3) unilateral upper limb movement disorder; (4) duration of stroke >6 months. The exclusion criteria were (1) severe cardiopulmonary and/or other organ dysfunction leading to unstable vital signs; (2) patients with psychiatric disorders or other neurological illnesses; (3) severe cognitive impairment or aphasia; (4) inability to complete the assessment; and (5) contraindications to TMS. All subjects were counseled about the purpose, content, and precautions of the study, and their written informed consent was obtained prior to enrolment in accordance with the Declaration of Helsinki. The study protocol was approved by the Ethics Committee of the Guangzhou First People's Hospital.

### 2.2 Motor Function Assessment

Before fNIRS data acquisition, upper limb motor function was assessed using the FMA scale. FMA is the most commonly used clinical measure for evaluating motor function with good consistency and accuracy.^17^ The scale is composed of 33 items, each of which is scored on a scale of 0–2; the total scores range from 0 to 66. The higher the FMA score, the better the motor function. Participants were divided into mild/moderate group (FMA > 30) and severe group (FMA≤30) based on the FMA score according to a previous study.^18^

### 2.3 fNIRS data acquisition and processing

The fNIRS data were acquired with a multichannel fNIRS system (Danyang Huichuang Medical Equipment Co. Ltd., China) using wavelengths of 730, 808, and 850 nm, with a sampling rate of 11 Hz. The emission and detector probes were placed on the head with regard to the international 10–20 system (distance between adjacent probes was 30 mm) and formed 63 measurement channels. Channels were localized and mapped to Brodmann area using a spatial registration approach in NirSpace. Recognizing the limitation that some patients are limited by motor impairment and are unable to complete specific motor tasks such as finger tapping or grasping, we designed our study as resting‐state assessments. During the 5‐min resting‐state recording, participants were instructed to close their eyes and stay relaxed but not fall asleep. The experiment was conducted in a quiet and semi‐dark room. After the data acquisition, fNIRS data processing and analysis were conducted offline by using Matlab (Mathworks, MA, USA) and Homer2.^19^ The raw light intensity data series were converted into optical density (OD) changes, and then motion artifacts were examined and corrected for the optical signals by the spline interpolation method. Bandpass filtering (0.01–0.1 Hz) was applied to the data using the Butterworth filter to eliminate physiological noise such as heartbeat and breathing. Subsequently, the OD data were transformed into hemoglobin concentration changes based on the modified Beer–Lambert law (MBLL).

In this study, we considered seven regions of interest (ROIs) that are known to be related to motor processing in both hemispheres: M1, PMC/SMA, primary somatosensory cortex (S1), somatosensory association cortex (SAC), dorsolateralprefrontal cortex (DLPFC), frontopolar cortex (FPC), and supramarginal gyrus (SMG). To evaluate within‐network connectivity, we calculated the Pearson's correlation coefficient for all ROI pairs in each subject and then Fisher transformation was applied to these coefficients.^20^ For the convenience of description, we made a flip on patients whose stroke locations were on the right side. Therefore, the left side was defined as the ispilesional side and the right side was defined as the contralesional side.

### 2.4 TMS assessment

In our study, the NS5000 Magnetic Stimulator (YIRUIDE Medical Co., Wuhan, China) with a figure‐of‐eight coil was employed to perform TMS assessments targeting MEP within the first dorsal interosseous (FDI) muscle, serving as an indicator of poststroke corticomotor pathway integrity. Participants maintained an open‐eye seated position while keeping their arms relaxed as instructed during the assessments. The coil placement during TMS involved positioning at a 45‐degree angle tangentially to the midline.^21^ The TMS recordings encompassed sequential measurements on both the unaffected and affected sides. The Visor2 neuronavigation system (Visor2, ANT Neuro, Hengelo, Netherlands) ensured precise coil localization, maintaining consistent stimulation over the FDI hotspot throughout the assessment. We first identified the motor hotspot on the unaffected hemisphere, defined as the optimal scalp position for eliciting maximal MEP amplitudes. Using the neuronavigation system, we then mirrored the motor hotspot to the corresponding area on the affected hemisphere consistent with previous study.^22^ TMS stimulation was applied to the mirrored point and its surrounding areas, with up to 10 single TMS pulses delivered at each stimulation point. Signal acquisition was performed at a sampling rate of 2000 Hz, with signal filtering tailored to a range of 3–1000 Hz. For MEP determination, MEP+ was defined as the induction of electromyography responses surpassing 50 microvolts in at least 5 out of 10 single‐pulse TMS stimulations. MEP− denoted the inability to elicit MEP, even at 100% of the maximum output intensity (MSO) during the assessment.^4^

### 2.5 Statistical analysis

Statistical analysis was performed using SPSS (SPSS, Chicago, IL, USA). The Shapiro–Wilk (SW) test was used to assess the normality of datasets. Independent samples *t*‐tests were applied for normally distributed continuous variables, while non‐parametric tests were used for variables that did not follow a normal distribution. For categorical variables such as sex, paretic side, type of stroke, and MEP status, the chi‐squared test was utilized. In this study, we compared a total of 106 features between the mild/moderate group and the severe group. These features included 14 oxygenated hemoglobin features derived from 14 ROIs, 91 FC features representing the connectivity patterns between the 14 ROIs, and 1 feature representing the MEP status. To control for Type I errors (false‐positives) resulting from these multiple comparisons, we applied the false discovery rate (FDR) correction to the *p*‐values obtained from the comparisons of the 106 features. Statistical significance was defined at *p* < 0.05, and all *p*‐values were two‐tailed.

### 2.6 Machine learning

Machine learning was performed on the Anaconda3 (www.anaconda.com) with Python (https://www.python.org/) and scikit‐learn package (scikit‐learn.org). To avoid dimensionality issues, variables displaying inter‐group differences were chosen to construct a model. The features were standardized to eliminate the differences between features and accelerate the convergence of model.^23^

**FIGURE 1:**
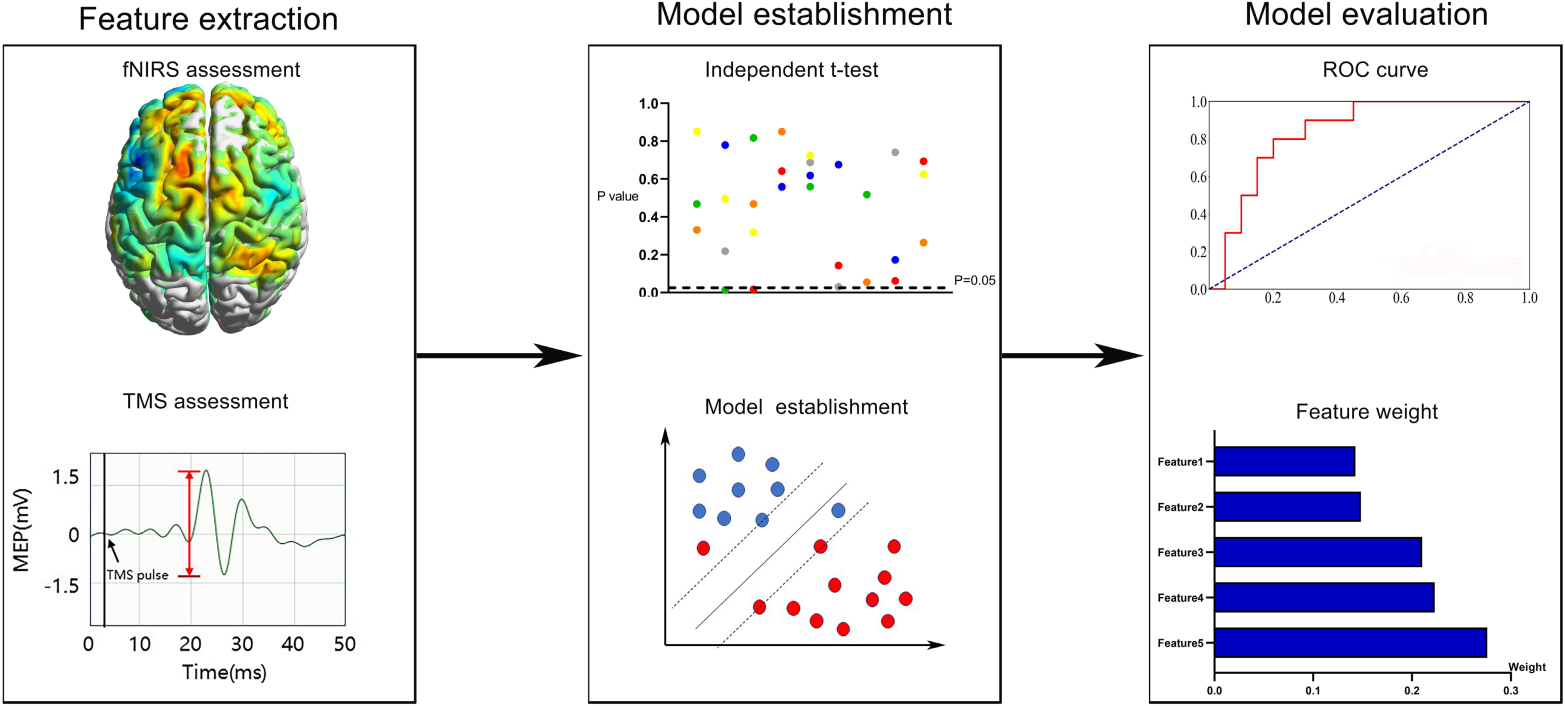
Flowchart of machine learning. First, the fNIRS and TMS assessments were conducted, followed by feature extraction. Subsequently, independent *t*‐test was applied to reduce the feature dimension, and machine learning model was developed with the retained features. Finally, the performance of model was evaluated by the AUC. fNIRS, functional near‐infrared spectroscopy; TMS, transcranial magnetic stimulation; AUC, area under the receiver operating characteristic curve.

In this study, four algorithms including random forest (RF), Gaussian Bayes, support vector machine (SVM), and logistic regression were applied to construct the prediction model based on the selected features. RF is an ensemble classifier which simultaneously builds several independent classification models and then makes the final decision through voting.^24^ Gaussian Bayes is a supervised learning classifier algorithm which classifies data points by probability statistics based on Bayes' theorem.^25^ The goal of SVM is to construct a decision hyperplane with the maximum distance to the nearest data points.^26^ Logistic regression is a statistical method specifically designed for predicting binary outcomes by estimating the probability of an input belonging to one of two categories. The dataset was subjected to leave‐one‐out cross‐validation (LOOCV) to ensure the generalizability of the machine learning models. In LOOCV, each data point was sequentially held out as a test case, while the remaining data were used for model training. This process was repeated for all data points, allowing us to assess the models' performance comprehensively. The performance of the predictive models was evaluated using accuracy and area under the receiver operating characteristic curve (AUC). To elevate the interpretability of ML models, shapley additive explanations (SHAP) values were calculated to quantify the weight of each variable in nonlinear models. The flowchart of machine learning is presented in Figure 1.

## 3 RESULTS

### 3.1 Demographic and clinical characteristics

Sixty patients were included in this study, comprising 33 individuals with mild/moderate hemiplegia (mean age: 61.00 ± 10.24; 24 males) and 27 patients with severe hemiplegia (mean age: 61.07 ± 9.33; 16 males). There were no statistically significant between‐group differences in terms of clinical characteristics, including age (*t* = 0.03, *p* = 0.98), sex (*χ* ^*2*^ = 1.21, *p* = 0.27), hemiplegia side (*χ* ^*2*^ = 0.13, *p* = 0.71), type (*p* = 0.52), and course of disease (*t* = 0.61, *p* = 0.55). Detailed demographic and clinical information for patients in both groups is provided in the Table 1.

**TABLE 1:** Patients' demographic and clinical characteristics.

	Age, years	Sex	Paretic side	Type of stroke	Months after stroke onset
	Mean ± SD (range)	Male/female	Right/left	Ischemic/hemorrhagic	Mean ± SD (range)
Mild/moderate group (*n* = 33)	61.00 ± 10.24 (36–76)	24/9	15/18	28/ 5	10.97 ± 4.18 (6–23)
Severe group (*n* = 27)	61.07 ± 9.33 (39–79)	16/11	11/16	21/ 6	11.52 ± 3.87 (6–17)
Statistical value	0.03	1.21	0.13	*	0.61
*p* value	0.98	0.27	0.71	0.52	0.55

*Note*: * indicates that no specific statistical value is provided by Fisher's exact test.

### 3.2 Comparisons of the two subgroups

**FIGURE 2:**
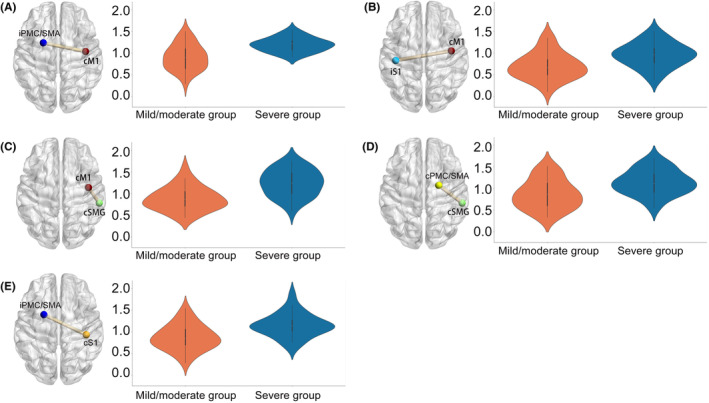
Violin plots showing the distribution of features selected for developing the machine learning models. (A) iPMC/SMA‐cM1, (B) iS1‐cM1, (C) cSMG‐cM1, (D) cSMG‐cPMC/SMA, (E) iPMC/SMA‐cS1. cM1, contralesional primary motor cortex; iSMA/PMC, ispilesional supplementary motor cortex/premotor cortex; cSMA/PMC, contralesional supplementary motor cortex/premotor cortex; iS1, ispilesional primary somatosensory cortex; cS1, contralesional primary somatosensory cortex; cSMG, contralesional supramarginal gyrus.

Patients with severe hemiplegia exhibited heightened FC in specific regions compared to those with mild to moderate hemiplegia, including iPMC/SMA‐cM1 (*t* = 4.56, *p_*_*FDR*_ <0.01, *Cohen's d* = 1.19), cSMG‐cM1 (*t* = 4.85, *p_*_*FDR*_ <0.01, *Cohen's d* = 1.26), cSMG‐cPMC/SMA (*t* = 3.36, *p_*_*FDR*_ = 0.02, *Cohen's d* = 0.87), iPMC/SMA‐cS1 (*t* = 3.85, *p_*_*FDR*_ = 0.01, *Cohen's d* = 1.00), and iS1‐cM1 (*t* = 3.40, *p_*_*FDR*_ = 0.02, *Cohen's d* = 0.88) (See Figure 2). Moreover, the incidence of MEP absence was significantly higher in patients with severe hemiplegia (81.48%) compared to those with mild to moderate hemiplegia (36.36%) (*χ* ^2^ = 12.31, *p_*_*FDR*_ = 0.01, *phi* = 0.46).

### 3.3 Performance of Machine Learning Models

**FIGURE 3:**
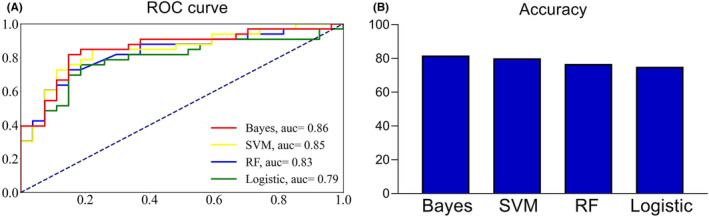
ROC curve and accuracy. (A) ROC curve: The ROC curve of different models. Bayes showed the highest AUC. (B) Accuracy: The accuracy of different models. Bayes showed the highest accuracy. ROC, receiver operating characteristic; AUC, area under the receiver operating characteristic curve; RF, random forest; SVM, support vector machine.

**FIGURE 4:**
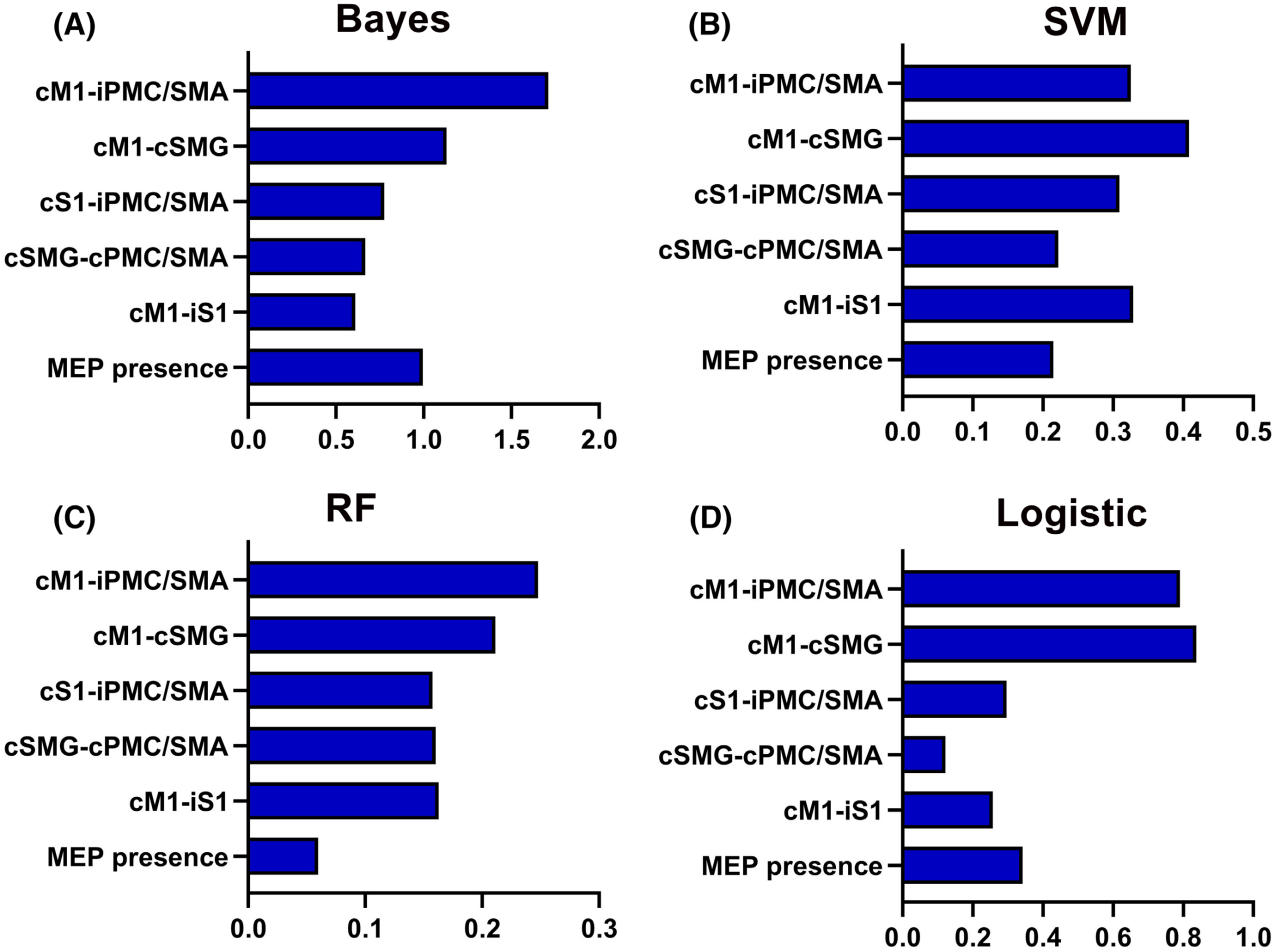
Histograms showing the weight of each feature in machine learning models. (A) Bayes, (B) SVM, (C) RF, (D) Logistic. RF, random forest; SVM, support vector machine.

Based on the results obtained from t‐tests and chi‐squared tests (refer to Section 3.2), a subset of six features was selected for constructing the machine learning models. Bayes demonstrated the highest classification accuracy at 81.67%, effectively distinguishing patients with mild/moderate hemiplegia from those with severe hemiplegia, achieving an AUC value of 0.86. SVM closely followed with an accuracy of 80.00% and an AUC of 0.85. Conversely, the RF and logistic models displayed comparatively lower predictive performance (See Figure 3 and Table 2). To explore the contribution of individual features to the models, we assessed feature importance. Notably, the FC of iPMC/SMA‐cM1 emerged as a significant contributor to the classification task, carrying the highest weight among the features in Bayes models (See Figure 4).

**TABLE 2:** Performance of the machine learning models.

	AUC	Accuracy	Recall	Precision
Bayes	0.856	81.67%	81.48%	81.81%
SVM	0.845	80.00%	81.48%	78.79%
RF	0.827	76.67%	74.07%	78.79%
Logistic	0.793	75.00%	74.07%	75.76%

Abbreviations: AUC, the area under the receiver operating characteristic curve; RF, random forest; SVM, support vector machine.

## 4 DISCUSSION

In this study, we revealed distinct FC patterns between mild/moderate and severe hemiplegia groups. Patients with severe hemiplegia displayed heightened connectivity in specific brain regions, indicating potential compensatory mechanisms or divergent reorganization strategies within the motor network. Moreover, machine learning was applied to make a distinction between the two subgroups. Among the evaluated models, Bayes showed the best performance with an accuracy of 81.67% and the connection of cM1‐iPMC/SMA made a substantial contribution to the classification. These results may enable a better understanding of the neural mechanisms supporting recovery among patients with different severity of motor impairment.

Our study highlights a notable enhancement in the connection of cM1 with other regions, including iPMC/SMA, iS1, and cSMG, in patients with severe hemiplegia. Indeed, the relationship between cM1 and poststroke motor recovery has been much debated. According to the interhemispheric competition model, the increased inhibition from cM1 to iM1 decreases the excitability of iM1, thereby hampering motor recovery.^27^ However, growing evidences showed that the interhemispheric competition model is oversimplified and may not be applicable to all stroke patients. In a recent study involving patients with severe hemiplegic stroke, elevation in excitation of cM1 with high‐frequency TMS led to improvement in motor function.^28^ Besides, neuroimaging studies have demonstrated that patients with severe hemiparesis are more likely to recruit more brain regions including cM1 to support motor function of the affected hand.^11^, ^29^ As has been described by previous study, the uncrossed corticospinal fibers from cM1 were found to innervate paretic muscles on the ispilesional side and supplement the damaged crossed corticospinal pathways in severely affected patients.^30^ Considering the above‐cited literature and our results, we speculate that cM1 may play an important role in brain reorganization in patients with severe hemiplegia.

In this study, we underscored the significance of iPMC/SMA in poststroke motor function. Stroke rehabilitation is a complex process that involves the coordination of multiple brain regions. Previous studies have demonstrated a significant involvement of secondary motor regions, such as the PMC and SMA, during motor tasks in stroke patients when compared to healthy controls.^29^ SMA is engaged in the initiation, learning, and planning of complex motor tasks.^31^ A previous study has shown that the representational map of the distal forelimb in the SMA may significantly enlarge after stroke and that the extent of expansion is directly proportional to the absolute size of the lesion.^32^ Further, researchers speculated that SMA may provide input to spinal motor neurons by involving theCST, thereby facilitating functional recovery.^30^ PMC is a key motor area for the planning and execution of hand movements. Researchers have demonstrated extensive projections of the PMC to M1 and spinal motor neurons, which may be an important factor for its engagement in motor recovery.^30^, ^33^ In a neuroimaging study, Rehme et al.^34^ found that the coupling of iSMA and iPMC with iM1 reduced after stroke and increased with recovery. Although previous studies and our findings have emphasized the significance of iPMC/SMA in patients with severe hemiplegia, the secondary motor areas exhibit reduced efficiency in generating motor output, thereby providing only partial compensation for the patient's motor function, rather than a complete restoration of function.^35^

A novel finding of our study was that enhanced connection of cM1‐iPMC/SMA in severe hemiplegia patients drove the classification. These results indicated distinct patterns of brain reorganization in patients with different severity of motor impairment. In a previous study, larger treatment gains after 3 weeks of standardized robotic therapy were found to be related to increased FC of iM1 with cPMC in patients with greater impairment, while the opposite relationship was observed in patients with less severe impairment.^36^ These results suggested that motor recovery in patients with severe stroke depends on the support of multiple brain regions, especially on the unaffected side, whereas in patients with less severe impairment, the recovery may rely on the restitution of normal circuitry. Moreover, Liu et al.^10^ found a positive relation of motor function with the FC of sensorimotor circuits in patients with partially paretic hand, whereas the relationship was negative in patients with completely paretic hand. This further reinforced the different functional reorganization for patients with different degrees of impairment. Furthermore, SMA and PMC have been shown to receive projections from the contralesional M1,^37^ and this may enable the coupling of cM1 with iSMA and PMC to promote the motor function recovery in patients with severe motor impairment. Collectively, we speculate that the enhanced FC of cM1‐iPMC/SMA in severely impaired patients may reflect a compensatory phenomenon, and this could explain its identification as a biomarker of the severity of hemiplegia.

The outcomes of this study revealed that the coupling of cSMG with cM1 contributed significantly to the classification models. SMG is situated in the inferior parietal lobule (IPL) and it is involved in attentiveness, motor function, and spatial perception.^38^ In a previous study, application of quadripulse transcranial magnetic stimulation over the SMG was found to decrease excitability of M1, indicating a significant interaction between the two areas.^39^ In a recent study, FMA scores showed a positive correlation with the effective connectivity from cSMG to the SMA, and the effective connectivity from cSMG to the ipsilesional superior frontal gyrus was identified as a biomarker for classifying patients with completely paretic hand and those with partially paretic hand.^40^ The results of this study in conjunction with those of the present study indicate that the enhanced coupling of cSMG and other functional areas may contribute to motor output of stroke patients, and it may serve as a new target for noninvasive stimulation.

Our findings highlight that the FC between S1 and other regions can serve as biomarkers for distinguishing between mild/moderate and severe hemiplegia in stroke patients. S1 is a crucial region in the cerebral cortex responsible for processing somatosensory information from various parts of the body. It provides continuous somatosensory input to M1, modulating its excitability and participating in synaptic plasticity processes.^41^ A previous study demonstrated that applying TMS stimulation to S1 improved motor function in stroke patients, underscoring the important role of S1 in motor recovery poststroke.^41^ Moreover, animal studies have shown that the contralesional M1 can establish stronger structural connections with the ipsilesional sensory and motor cortical regions in cases of large infarcts, and these structural changes are closely related to motor function recovery.^42^ This suggests that the contralesional hemisphere may compensate for the loss of the ipsilesional hemisphere by reshaping neuronal circuits and establishing new sensory processing pathways. Interestingly, a recent study by Williamson et al.^43^ reported a hemispheric shift in sensory processing after stroke, with bilateral activation of S1 in response to tactile stimulation of the paretic hand in stroke patients, while healthy controls showed only contralateral S1 activation. This finding is consistent with our results, indicating a higher degree of reliance on the contralesional hemisphere for sensory processing in severely impaired patients compared to those with mild to moderate impairment.

Our study revealed a significantly higher proportion of absent MEP in patients with severe hemiplegia compared to those with mild to moderate impairment. Interestingly, recent evidence suggests that absent MEP does not always indicate poor functional recovery.^5^ This discrepancy might be explained by the increased reliance on contralesional indirect motor pathways in severe hemiplegia. Previous research has demonstrated a gradual shift of brain activity toward the contralesional hemisphere as the motor demand on the affected side increases.^44^ This implies that when the ipsilesional CST is substantially damaged, patients may recruit alternative pathways in the contralesional hemisphere to maintain motor function. Williamson et al. showed that transcranial direct current stimulation over the contralesional PMd can modulate the excitability of the ipsilesional M1, indicating a structural or functional connection between these areas, which may serve as a basis for the contralesional hemisphere to support the ipsilesional hemisphere.^45^ Furthermore, electrophysiological measures, such as brain–muscle connectivity (BMC), have shown increased nonlinear connectivity in the contralesional hemisphere with higher motor demand,^46^ supporting the notion of increased reliance on indirect pathways poststroke. Taken together, we propose that although patients with severe hemiplegia generally have poorer functional recovery, they may engage contralesional indirect pathways as a compensatory strategy to maintain some degree of motor function. Future investigations could integrate MEP, BMC, and other electrophysiological indicators to elucidate the dynamic reorganization of motor pathways in severe stroke and its association with functional recovery.

In our study, we explored the potential differences in clinical factors such as age, sex, and stroke type between the mild/moderate and severe hemiplegia groups. However, no significant differences were observed in these demographic and clinical characteristics. Notably, while we did not find significant sex differences in our cohort, some previous studies have suggested potential sex‐related differences in poststroke recovery.^47^ Ryu et al.^48^ reported that women with cortical infarcts, especially in the left parieto‐occipital regions, had higher National Institutes of Health Stroke Scale (NIHSS) scores compared to men, indicating that lesions in certain brain regions may have a greater impact on function in women. However, other studies found that motor recovery and functional outcomes after stroke were not influenced by sex. Dahlby et al.^49^ reported no significant differences between males and females in objective and subjective measures of upper limb motor function. Similarly, Stinear et al.^50^ found that recovery from motor impairment was unaffected by sex, and instead was proportional to initial impairment. Given the inconsistencies in the literature, further research with larger, well‐characterized cohorts and advanced neuroimaging techniques is warranted to clarify whether sex plays a role in neural reorganization and recovery after stroke. Such studies may elucidate potential sex differences in the neural mechanisms of motor recovery, informing the development of individualized rehabilitation strategies.

This study contributes significantly to a deeper comprehension of the neural aspects linked to motor function after a stroke. By categorizing patients into mild/moderate and severe hemiplegia subgroups, we were able to precisely compare brain connectivity patterns across different levels of severity, potentially aiding functional recovery strategies. Employing machine learning for subgroup identification marks a crucial advancement toward data‐centric prognosis and personalized interventions. If distinct neural markers, such as heightened connectivity between bilateral hemispheric motor areas, prove beneficial for motor function, noninvasive brain stimulation methods like TMS could effectively modulate these pathways, enhancing neurorehabilitation outcomes. Constructing predictive models based on neuroimaging metrics is pivotal for obtaining insights that can be practically applied, potentially assisting clinicians in targeting specific network connections affected in individual patients using tailored stimulation approaches. This research not only progresses our theoretical models of stroke recovery but also guides future endeavors aimed at harnessing neuromodulation techniques to improve prognostic assessments and therapeutic responses across different patient subgroups.

There were some limitations in this study. First, the cross‐sectional study design precludes causal inferences between reorganization of brain networks during motor recovery. Additionally, healthy controls were not included due to recruitment difficulties of well‐matched subjects (in terms of age, gender, comorbidities, etc.). However, as this study primarily focused on stratifying chronic stroke subgroups, the lack of controls did not compromise the interpretations or conclusions concerning impairment severity. Nonetheless, future studies involving healthy subjects could provide further comparative insights. Moreover, despite the large effect sizes observed in the current study, future research with larger sample sizes is warranted to validate and extend our findings.

## 5 CONCLUSION

In summary, our research has leveraged machine learning models to pinpoint potential biomarkers linked to motor function in stroke patients. The outcomes of our investigation have illuminated unique brain network reorganization patterns within patients exhibiting varying degrees of motor impairment. These findings contribute to an enriched comprehension of the mechanisms underlying motor function recovery, promising future developments in therapies that aim to enhance neural processes associated with motor rehabilitation.

## AUTHOR CONTRIBUTIONS

**Guiyuan Cai:** Performing research; analyzing data; and writing draft of paper. **Jiayue Xu:** Performing research; analyzing data: and editing and reviewing paper. **Cailing Zhang**: Performing research and methodology. **Junbo Jiang:** Performing research and curating data. **Gengbin Chen:** Performing research and curating data. **Jialin Chen:** Methodology and formal analysis. **Quan Liu:** Recruiting participants. **Guangqing Xu:** Designing research; methodology; and acquiring financial support. **Yue Lan:** Designing research; editing; and reviewing paper; and acquiring financial support.

## FUNDING INFORMATION

This work was supported by grant 2022YFC2009700 from Natural Key Research and Development Program of China (to Yue Lan), grant 81974357 from National Science Foundation of China (to Yue Lan), grant 202206010197 and 202201020378 from Guangzhou Municipal Science and Technology Program (to Yue Lan), and grant 82072548 from National Science Foundation of China (to Guangqing Xu).

## CONFLICT OF INTEREST STATEMENT

None of the authors have potential conflicts of interest to be disclosed.

## CONSENT FOR PUBLICATION

Written informed consent for publication was obtained from all subjects.

## Data Availability

The datasets are available by request from the corresponding author.
